# Pervasive Sharing of Genetic Effects in Autoimmune Disease

**DOI:** 10.1371/journal.pgen.1002254

**Published:** 2011-08-10

**Authors:** Chris Cotsapas, Benjamin F. Voight, Elizabeth Rossin, Kasper Lage, Benjamin M. Neale, Chris Wallace, Gonçalo R. Abecasis, Jeffrey C. Barrett, Timothy Behrens, Judy Cho, Philip L. De Jager, James T. Elder, Robert R. Graham, Peter Gregersen, Lars Klareskog, Katherine A. Siminovitch, David A. van Heel, Cisca Wijmenga, Jane Worthington, John A. Todd, David A. Hafler, Stephen S. Rich, Mark J. Daly, on behalf of the FOCiS Network of Consortia

**Affiliations:** 1 Center For Human Genetic Research, Massachusetts General Hospital, Boston, Massachusetts, United States of America; 2 Medical and Population Genetics, Broad Institute of MIT and Harvard, Cambridge, Massachusetts, United States of America; 3 Department of Medicine, Harvard Medical School, Boston, Massachusetts, United States of America; 4 Department of Neurology, Yale University School of Medicine, New Haven, Connecticut, United States of America; 5 Department of Genetics, Yale University School of Medicine, New Haven, Connecticut, United States of America; 6 Health Science and Technology MD Program, Harvard University and Massachusetts Institute of Technology, Boston, Massachusetts, United States of America; 7 Harvard Biological and Biomedical Sciences Program, Harvard University, Boston, Massachusetts, United States of America; 8 Pediatric Surgical Research Laboratories, Massachusetts General Hospital, Boston, Massachusetts, United States of America; 9 Center for Biological Sequence Analysis, Department of Systems Biology, Technical University of Denmark, Lyngby, Denmark; 10 Analytical and Translational Genetics Unity, Massachusetts General Hospital, Boston, Massachusetts, United States of America; 11 Juvenile Diabetes Research Foundation/Wellcome Trust Diabetes and Inflammation Laboratory, Department of Medical Genetics, Cambridge Institute for Medical Research, University of Cambridge, Cambridge, United Kingdom; 12 Center for Statistical Genetics, University of Michigan, Ann Arbor, Massachusetts, United States of America; 13 Human Genetics, Wellcome Trust Sanger Institute, Cambridge, United Kingdom; 14 Genentech, South San Francisco, California, United States of America; 15 Departments of Medicine and Genetics, Yale University School of Medicine, New Haven, Connecticut, United States of America; 16 Department of Neurology, Brigham and Women's Hospital, Boston, Massachusetts, United States of America; 17 Department of Dermatology, University of Michigan, Ann Arbor, Michigan, United States of America; 18 Feinstein Institute for Medical Research, North Shore-Long Island Jewish Health System, Manhasset, New York, United States of America; 19 Rheumatology Unit, Department of Medicine, Karolinska Institutet, Stockholm, Sweden; 20 Samuel Lunenfeld Research Institute, Mount Sinai Hospital, Toronto, Canada; 21 Blizard Institute, The London School of Medicine and Dentistry, London, United Kingdom; 22 Department of Genetics, University Medical Center Groningen and Groningen University, Groningen, The Netherlands; 23 Arthritis Research UK Epidemiology Unit, School of Translational Medicine, Manchester Academic Health Sciences Centre, University of Manchester, Manchester, United Kingdom; 24 Center for Public Health Genomics, University of Virginia, Charlottesville, Virginia, United States of America; University of Geneva Medical School, Switzerland

## Abstract

Genome-wide association (GWA) studies have identified numerous, replicable, genetic associations between common single nucleotide polymorphisms (SNPs) and risk of common autoimmune and inflammatory (immune-mediated) diseases, some of which are shared between two diseases. Along with epidemiological and clinical evidence, this suggests that some genetic risk factors may be shared across diseases—as is the case with alleles in the Major Histocompatibility Locus. In this work we evaluate the extent of this sharing for 107 immune disease-risk SNPs in seven diseases: celiac disease, Crohn's disease, multiple sclerosis, psoriasis, rheumatoid arthritis, systemic lupus erythematosus, and type 1 diabetes. We have developed a novel statistic for Cross Phenotype Meta-Analysis (CPMA) which detects association of a SNP to multiple, but not necessarily all, phenotypes. With it, we find evidence that 47/107 (44%) immune-mediated disease risk SNPs are associated to multiple—but not all—immune-mediated diseases (SNP-wise *P*_CPMA_<0.01). We also show that distinct groups of interacting proteins are encoded near SNPs which predispose to the same subsets of diseases; we propose these as the mechanistic basis of shared disease risk. We are thus able to leverage genetic data across diseases to construct biological hypotheses about the underlying mechanism of pathogenesis.

## Author Summary

Over the last five years we have found over 100 genetic variants predisposing to common diseases affecting the immune system. In this study we analyze 107 such variants across seven diseases and find that almost half are shared across diseases. We also find that the patterns of sharing across diseases cluster these variants into groups; proteins encoded near variants in the same group tend to interact. This suggests that genetic variation may influence entire pathways to create risk to multiple diseases.

## Introduction

The human immune-mediated diseases are the result of aberrant immune responses. These immune responses may lead to chronic inflammation and tissue destruction, often targeting a specific organ site. The outcome of this process is immune-mediated inflammatory and autoimmune disease, affecting approximately 5% of the population [1].

Extensive clinical and epidemiologic observations have shown that immune-mediated inflammatory and autoimmune diseases can occur either in the same individual or in closely related family members. This clustering of multiple diseases appears more frequently than expected if disease processes were independent. As each of the immune-mediated inflammatory and autoimmune diseases has strong genetic influences on disease risk [2]–[7], the observed clustering of multiple diseases could be due to an overlap in the causal genes and pathways [8], [9].

The patterns of clustering of diseases across the population are complex [10] – each disease has a prevalence between 0.01%–3%, so direct assessment of co-aggregation within individuals or families does not result in the very large samples required for genetic or epidemiological investigation. Thus it is unsurprising that to date, these observations have yet to be translated into determinants of the shared molecular etiologies of disease.

Recent GWA studies in immune-mediated and autoimmune diseases have identified 140 regions of the genome with statistically significant and robust evidence of presence of disease susceptibility loci. A subset of these loci have been shown to modulate risk of multiple diseases [3], [6], [11]–[14]. In addition, there is evidence that loci predisposing to one disease can have effects on risk of a second disease [15], although the risk allele for one disease may not be the same as for the second [16].

Together, these observations support the hypothesis of a common genetic basis of immune-mediated and autoimmune diseases [17]. There is now the ability to estimate both the number of loci contributing to risk of multiple diseases and the spectrum of diseases that each locus influences. In addition, grouping variants by the diseases they influence should provide insight into the specific biological processes underlying co-morbidity and disease risk.

In this report, we systematically investigate the genetic commonality in immune-mediated inflammatory and autoimmune diseases by examining the contributions of associated genomic risk regions in seven diseases: celiac disease (CeD), Crohn's disease (CD), multiple sclerosis (MS), psoriasis (Ps), rheumatoid arthritis (RA), systemic lupus erythematosus (SLE) and type 1 diabetes (T1D). We find that nearly half of loci identified in GWAS studies of an individual disease influence risk to at least two diseases, arguing for a genetic basis to co-morbidity. We also find several variants with opposing risk profiles in different diseases. Supporting the idea that common patterns of association implicate shared biological processes, we further demonstrate that loci clustered by the pattern of diseases they affect harbor genes encoding interacting proteins at a much higher rate than by chance. These results suggest that multi-phenotype mapping will identify the molecular mechanisms underlying co-morbid immune-mediated inflammatory and autoimmune diseases.

## Results

We first test our hypothesis of common genetic determinants by examining evidence of association of genetic variants in known immune-mediated and autoimmune disease susceptibility loci to multiple disease phenotypes. We collated a list of 140 single nucleotide polymorphisms (SNPs) representing reported associations to at least one immune-mediated disease at genome-wide significance levels. Where data for the reported SNP itself were not available in our GWA studies (Table 1), we chose a proxy in high linkage disequilibrium to the reported marker (*r^2^*>0.9 in HapMap/CEU). We did not consider SNPs in the human Major Histocompatibility Complex (MHC) from this analysis, as its role in many of these diseases is well-established and the classically associated alleles in the HLA region are not well captured by SNPs [18]. We were able to acquire data for either the reported SNP or a good proxy in 107 of 140 cases, and assembled genotype test summaries for these from previously described GWA studies representing over 26,000 disease cases (Table 1).

**Table 1:** Participating studies.

Disease	Cases	Controls	Reference
Celiac disease	3796	8154	22
Crohn's disease	3230	4829	1
Multiple sclerosis	2624	7220	4
Psoriasis	1359	1400	5
Rheumatoid arthritis	5539	20169	6
Systemic Lupus Erythematosus	1963	4329	23
Type 1 diabetes	7514	9045	24

Data were collated for seven phenotypes from meta-analyses incorporating all known genome-wide association studies. SLE is the exception as no comprehensive meta-analysis has yet been published; data were instead obtained from a recent meta-analysis including some, but not all, known genome-wide association studies. Note that controls overlap in some cases due to the use of common shared sample genotypes.

We have developed a cross-phenotype meta-analysis (CPMA) statistic to assess association across multiple phenotypes. The CPMA statistic determines evidence for the hypothesis that each independent SNP has multiple phenotypic associations. Support for this hypothesis would be shown by deviations from expected uniformity of the distribution of association *p*-values, indicative of multiple associations. The likelihood of the observed rate of exponential decay of −log_10_(*p*) is calculated and compared to the null expectation (the decay rate should be unity) as a likelihood ratio test (see Materials and Methods for details). This CPMA statistic has one degree of freedom, as it measures a deviation in *p*-value behavior instead of testing all possible combinations of diseases for association to each SNP.

A total of 47 of the 107 SNPs tested have evidence of association to multiple diseases (SNP-wise *P_CPMA_*<0.01; expectation roughly 1 by chance; binomial probability of observing this result *p* = 3×10^−64^). This highly significant result confirms widespread sharing of genetic loci between immune-mediated inflammatory and autoimmune diseases. Further, these “multi-phenotype” SNPs include many loci not previously known to be shared across diseases, as well as new predictions of association for previously known shared loci (Table 2).

**Table 2:** SNPs associated with multiple phenotypes.

SNP						RA		Psoriasis		MS		SLE		Crohn		Coeliac		T1D		CPMA	Reference
Name	Chr	Position	Aj	Am	Genes	Z	*p*	Z	*p*	Z	*p*	Z	*p*	Z	*p*	Z	*p*	Z	*p*	*p*	
rs10889677	1	67437141	C	A	IL23R	0.0	9.8E-01	5.1	3.5E-07	−2.6	4.9E-03	−0.3	6.2E-01	10.3	9.0E-25	1.1	8.7E-01	0.1	5.3E-01	6.9E-25	37
rs3087243	2	204564425	G	A	CTLA4	−5.7	1.2E-08	0.3	8.0E-01	−0.7	2.5E-01	−0.5	3.0E-01	−1.2	2.3E-01	2.9	2.2E-03	−8.5	1.1E-17	2.8E-21	36
rs2542151	18	12769947	T	G	PTPN2	4.2	3.0E-05	2.0	4.1E-02	−0.8	2.2E-01	1.1	1.5E-01	6.8	1.2E-11	4.8	6.9E-07	7.1	5.9E-13	5.3E-19	1
rs2201841	1	67406223	A	G	IL23R	0.0	9.9E-01	5.2	2.7E-07	−2.2	1.3E-02	−0.3	6.3E-01	9.9	3.5E-23	1.0	8.4E-01	0.2	5.7E-01	3.7E-18	5
rs11209032	1	67452113	G	A	IL23R	−0.6	5.2E-01	5.3	1.3E-07	−1.2	1.2E-01	−0.7	2.3E-01	8.4	3.1E-17	1.2	8.8E-01	0.2	4.4E-01	6.4E-18	33
rs1893217	18	12799340	A	G	PTPN2	4.2	2.4E-05	2.1	3.9E-02	−0.6	2.8E-01	1.0	1.5E-01	6.5	6.5E-11	4.9	6.1E-07	7.4	8.2E-14	3.4E-17	24
rs917997	2	102529086	C	T	IL18RAP	0.3	7.8E-01	−0.2	8.8E-01	0.3	3.7E-01	1.1	1.3E-01	4.2	2.2E-05	7.6	1.1E-14	−1.5	9.4E-01	4.9E-13	3
rs12708716	16	11087374	A	G	CLEC16A	0.2	8.3E-01	−0.3	8.1E-01	3.7	1.1E-04	−1.0	1.5E-01	−0.6	5.5E-01	1.6	5.3E-02	−8.2	1.2E-16	2.0E-12	32
rs2872507	17	35294289	G	A	ORMDL3	4.1	4.7E-05	0.3	8.0E-01	−3.3	5.5E-04	−0.3	6.3E-01	4.7	2.1E-06	0.7	7.6E-01	5.0	2.5E-07	4.1E-09	1
rs3821236	2	191728264	G	A	STAT4	4.7	2.5E-06	−1.7	9.5E-02	0.1	4.6E-01	6.2	2.1E-10	−1.7	9.6E-02	1.9	3.1E-02	3.4	3.5E-04	9.8E-08	2
rs6441961	3	46327388	C	T	CCR1	1.1	2.7E-01	−0.3	7.5E-01	0.1	4.7E-01	1.3	9.1E-01	−0.5	6.1E-01	5.7	6.9E-09	3.7	1.0E-04	1.9E-07	3
rs2290400	17	35319766	C	T	ORMDL3	−3.9	1.1E-04	0.4	7.2E-01	3.3	5.5E-04	0.2	5.7E-01	3.7	2.4E-04	0.2	4.1E-01	−5.1	1.4E-07	1.2E-06	24
rs7197475	16	30550368	C	T	16p11.2	2.9	3.8E-03	2.9	4.3E-03	-0.9	1.7E-01	0.7	2.4E-01	−4.2	2.5E-05	0.4	3.4E-01	−0.9	1.9E-01	2.0E-06	35
rs4917014	7	50083124	T	G	IKZF1	−2.5	1.3E-02	0.9	3.8E-01	3.5	2.6E-04	−2.9	1.9E-03	1.6	1.1E-01	0.9	1.8E-01	−3.3	5.2E-04	4.9E-06	35
rs6822844	4	123867026	G	T	IL2-IL21	−3.4	6.5E-04	−1.7	8.7E-02	1.2	1.1E-01	-0.2	4.3E-01	−2.4	1.5E-02	3.3	4.9E-04	−1.1	1.5E-01	6.2E-06	38
rs10517086	4	25761780	G	A	4p15.2	5.1	2.8E-07	0.6	5.7E-01	0.4	3.6E-01	0.1	5.5E-01	2.8	5.5E-03	0.2	5.6E-01	4.9	5.7E-07	2.3E-05	24
rs11203203	21	42709255	G	A	UBASH3A	4.2	2.5E-05	1.3	1.9E-01	0.2	4.4E-01	1.6	5.3E-02	0.0	9.8E-01	3.1	1.0E-03	6.6	1.8E-11	2.5E-05	24
rs4728142	7	128167918	G	A	IRF5	4.5	7.1E-06	1.4	1.8E-01	−1.5	6.4E-02	6.2	2.9E-10	0.8	4.4E-01	2.1	1.6E-02	−0.2	4.1E-01	4.4E-05	35
rs11755527	6	91014952	C	G	BACH2	−1.4	1.6E-01	−0.7	5.1E-01	−2.8	2.7E-03	1.0	8.5E-01	−2.6	1.0E-02	3.5	2.8E-04	5.6	1.0E-08	8.0E-05	24
rs7709212	5	158696755	T	C	IL12B	−0.6	5.7E-01	−6.3	3.8E-10	3.6	1.9E-04	−2.3	1.1E-02	3.3	9.8E-04	0.1	5.5E-01	1.2	8.9E-01	8.8E-05	39
rs947474	10	6430456	A	G	PRKCQ	−4.4	8.7E-06	−1.0	3.4E-01	−0.3	6.3E-01	−1.0	8.5E-01	−2.4	1.9E-02	2.0	2.1E-02	−3.7	1.1E-04	9.1E-05	34
rs2188962	5	131798704	C	T	5q31	−0.3	7.7E-01	3.0	3.2E-03	1.3	9.6E-02	1.5	7.0E-02	5.9	4.6E-09	1.8	3.7E-02	2.9	2.0E-03	1.6E-04	1
rs744166	17	37767727	A	G	STAT3	−0.7	4.7E-01	1.3	1.9E-01	-4.4	6.4E-06	0.7	7.6E-01	−4.5	5.9E-06	2.2	9.8E-01	−2.6	4.6E-03	2.2E-04	1
rs4788084	16	28447349	C	T	IL27	2.6	1.0E-02	1.3	2.1E-01	0.3	6.1E-01	1.3	9.1E-02	2.9	3.5E-03	2.3	1.1E-02	−6.5	5.1E-11	4.1E-04	24
rs2082412	5	158650367	G	A	IL12B	−1.2	2.3E-01	−6.2	8.8E-10	NA	NA	−3.8	7.1E-05	2.6	9.6E-03	0.4	3.6E-01	0.0	5.0E-01	4.6E-04	5
rs11465804	1	67414547	T	G	IL23R	−0.4	6.8E-01	−4.9	1.3E-06	−1.7	9.5E-01	−0.4	3.6E-01	−12.5	1.0E-35	1.2	1.2E-01	−1.0	1.6E-01	5.2E-04	1
rs463426	22	20133739	C	T	HIC2-UBE2L3	2.2	2.9E-02	1.4	1.6E-01	1.0	1.7E-01	0.3	3.7E-01	1.8	6.7E-02	3.3	4.1E-04	0.5	2.9E-01	5.2E-04	35
rs763361	18	65682622	C	T	CD226	2.1	3.3E-02	1.9	6.5E-02	−1.7	4.5E-02	0.0	4.9E-01	2.0	4.1E-02	2.5	6.7E-03	5.1	1.6E-07	9.0E-04	24
rs11584383	1	197667523	T	C	KIF21B	2.3	2.3E-02	0.0	9.7E-01	3.3	4.6E-04	−0.3	6.2E-01	−5.0	6.8E-07	0.8	2.0E-01	−2.3	1.0E-02	9.0E-04	1
rs6590330	11	127816269	G	A	ETS1	3.2	1.5E-03	−0.5	6.0E-01	−2.7	3.7E-03	2.1	1.9E-02	0.6	5.2E-01	1.4	9.2E-01	1.8	3.9E-02	2.6E-03	35
rs4900384	14	97568704	A	G	14q32.2	1.3	2.0E-01	0.8	4.4E-01	1.8	3.9E-02	−1.9	3.0E-02	0.2	8.1E-01	3.2	7.6E-04	5.2	8.9E-08	2.8E-03	24
rs10758669	9	4971602	A	C	JAK2	1.0	3.3E-01	1.2	2.4E-01	−3.3	4.2E-04	0.3	4.0E-01	5.0	6.8E-07	1.8	3.7E-02	1.0	1.7E-01	3.4E-03	1
rs1913517	10	49789060	A	G	LRRC18-WDFY4	−4.4	1.1E-05	0.8	4.4E-01	0.4	3.5E-01	−2.4	8.7E-03	−0.4	6.9E-01	0.1	4.4E-01	-0.8	2.2E-01	4.1E-03	35
rs4505848	4	123490097	A	G	IL2	−0.5	6.0E-01	0.1	9.0E-01	0.5	7.0E-01	1.1	1.3E-01	3.0	2.6E-03	3.1	9.6E-04	6.6	2.3E-11	4.4E-03	24
rs7804356	7	26664905	T	C	7p15.2	−1.3	1.9E-01	1.2	2.2E-01	1.0	1.5E-01	−0.1	4.8E-01	3.4	5.9E-04	1.3	9.9E-02	−5.6	9.5E-09	5.6E-03	24
rs11258747	10	6512897	G	T	PRKCQ	3.0	2.4E-03	1.9	6.0E-02	-2.5	6.7E-03	0.6	7.3E-01	0.1	9.2E-01	0.2	4.2E-01	4.8	7.7E-07	7.5E-03	24
rs703842	12	56449006	A	G	CYP27B1	−2.7	6.1E-03	−0.1	8.9E-01	4.1	1.7E-05	−0.4	6.7E-01	−1.1	2.5E-01	2.0	2.3E-02	−2.2	1.5E-02	8.3E-03	31
rs1990760	2	162949558	T	C	IFIH1	−1.0	3.0E-01	−2.4	1.7E-02	0.1	5.4E-01	−3.4	3.2E-04	0.6	5.6E-01	0.4	6.5E-01	−6.2	2.5E-10	8.6E-03	24
rs2476601	1	114089610	G	A	PTPN22	18.2	9.1E-74	0.0	1.0E+00	0.4	3.5E-01	4.0	3.3E-05	−4.3	1.8E-05	1.7	4.2E-02	20.4	1.5E-92	6.3E-160	1
rs3184504	12	110347328	T	C	SH2B3	−2.9	3.6E-03	−2.0	4.1E-02	3.4	3.3E-04	−2.7	3.6E-03	−3.4	6.2E-04	7.3	1.2E-13	−11.9	7.7E-33	4.3E-19	24
rs11865121	16	11074189	C	A	CLEC16A	0.3	7.7E-01	0.6	5.2E-01	4.3	8.7E-06	−0.7	2.3E-01	0.9	3.8E-01	1.1	1.4E-01	−8.9	2.1E-19	1.1E-14	4
rs2816316	1	189268470	A	C	RGS1	−1.0	3.3E-01	−0.4	6.7E-01	3.1	9.0E-04	−0.1	5.3E-01	−0.5	6.4E-01	6.9	2.7E-12	−3.9	4.2E-05	5.2E-13	3
rs2104286	10	6139051	T	C	IL2RA	−3.1	1.8E-03	−0.1	9.5E-01	6.2	3.5E-10	0.4	6.5E-01	−0.8	4.4E-01	0.5	3.1E-01	−6.4	5.9E-11	1.2E-08	32
rs3024505	1	203328299	G	A	IL10	−1.0	3.3E-01	0.9	3.8E-01	1.5	7.3E-02	4.2	1.3E-05	2.4	1.6E-02	1.6	5.4E-02	−4.8	6.2E-07	2.2E-06	23
rs10045431	5	158747111	C	A	IL12B	0.4	6.5E-01	4.5	6.6E-06	0.3	6.3E-01	2.4	8.8E-03	−5.8	8.8E-09	0.5	6.8E-01	0.1	4.6E-01	6.0E-04	1
rs610604	6	138241110	T	G	TNFAIP3	−4.2	3.3E-05	4.5	8.0E-06	0.3	3.8E-01	−1.3	9.9E-02	1.4	1.8E-01	0.4	6.5E-01	−0.2	4.3E-01	2.7E-03	5
rs4613763	5	40428485	T	C	PTGER4	0.9	3.9E-01	0.7	4.7E-01	−4.2	1.1E-05	0.4	3.4E-01	9.6	5.0E-22	0.1	5.2E-01	−0.5	3.1E-01	4.0E-03	1

47/107 SNPs tested showed significant evidence of association to multiple diseases (*P_cpma_*<0.01), where only one is expected by chance. These SNPs are therefore candidate drivers for the shared genetic architecture between diseases. The SNPs shown in the lower panel also have strong evidence of association in opposite directions across phenotypes and may be crucial decision points in pathogenesis. *Aj = major allele; Am = minor allele*. Z scores are reported from published GWA studies and arbitrarily signed relative to the direction of effect in celiac disease. Note that no MS data were available for rs2082412 as it had not been imputed accurately in the participating MS study. Data for all SNPs is presented in Dataset S1.

Although our CPMA statistic is agnostic to effect direction in each disease, a subset of the 47 multi-phenotype (CPMA positive) SNPs appeared to have strong allelic effects in opposite directions in different diseases [16]. A total of 9 SNPs had strong evidence of such directional association (an association *p*<1×10^−4^ with at least one protective and one risk effect; lower panel in Table 2). This suggests that shared associations have complex effects on disease outcomes and may be of particular importance in pathogenic processes.

We next examined the patterns of association across 47 multi-phenotype SNPs to determine evidence of either a global autoimmune process or biological pathways influencing sets of diseases. On visual inspection of these data we found a striking patterning of associations across diseases: only one SNP (rs3184504, in an exon of *SH2B3*) exhibited evidence of association to all seven diseases; the others appeared to associate only to subsets of diseases (Table 2).

**Figure 1:**
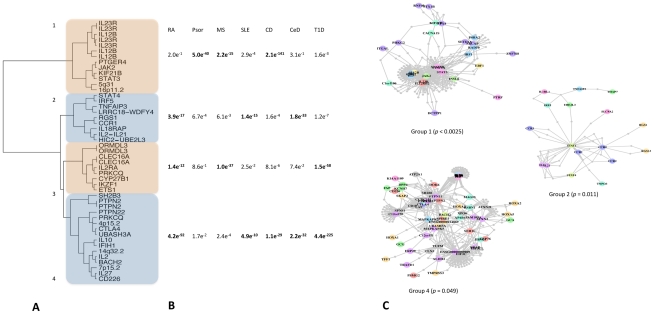
Patterns of association across diseases correlate with protein-protein interactions. A: 47 SNPs with evidence of association to multiple diseases (*P_cpma_*<0.01) fall into groups clustered by the pattern of association across diseases. Clusters are numbered arbitrarily. B: Clusters show different patterns of association across diseases. We summarize the differential disease effects of each cluster with a cumulative association statistic (Fisher's method for combining *p* values). These patterns are different for each cluster, suggesting each represents a different co-morbid mechanism. Note that these figures are based on the same underlying association statistics the clustering in the first panel is derived from. C: proteins encoded within the linkage disequilibrium scope around SNPs in the same cluster interact either directly or via common intermediates. Three of our four clusters have significant protein inter-connectivity (permuted *P*<0.05; see Materials and Methods and [19] for details).

To formalize the analysis of association patterns across diseases, we determined specific patterns of associations across SNPs by computing SNP-SNP distances based on the level of association to each disease followed by hierarchical clustering to group them (Figure 1A; see Materials and Methods section for clustering details). SNPs in loci encoding proteins known to interact clustered together: for example, the independent effects at *IL12B* and *IL23R*, which encode subunits of a ligand-receptor pair are in the same region of the dendrogram. We next partitioned the dendrogram “tree” into four clusters and summarized the cumulative association of each cluster to each disease by combining our underlying dataset of association *p*-values per cluster, per disease using Fisher's omnibus test (Figure 1B; see Materials and Methods for details). Each cluster had a different pattern of associations across diseases; these patterns suggest that the clusters represent distinct co-morbid mechanisms.

Our underlying hypothesis has been that phenotype-driven clusters represent distinct molecular mechanisms. This leads to the prediction that components of these clusters/pathways are encoded in associated loci; in other words, proteins encoded around SNPs in the same cluster should interact. We test this prediction by looking for connectivity between proteins encoded around SNPs within each cluster as described elsewhere [19]. Briefly, we define a genomic region around each SNP in terms of linkage disequilibrium and consider any protein overlapping that region. We then ask if proteins encoded around SNPs in the same cluster interact using protein-protein interaction maps, excluding interactions between proteins in the same region (see Materials and Methods and [19]). We find that three of the four clusters we define by patterns of association have significant connectivity (Figure 1C; permuted *P*<0.05) by this method, suggesting that these represent distinct molecular mechanisms affected by genetic risk variants. Two of these groups of interacting proteins are also preferentially expressed [19] in immune cell subtypes compared to other tissue types (Figure S1), supporting our hypothesis that these represent true pathways underlying pathogenesis.

## Discussion

Immune-mediated inflammatory and autoimmune diseases have been known to cluster in families, suggesting a strong genetic component to risk. The genes in the human MHC (HLA complex) have been associated with disease risk, suggesting a common immune pathway. Less clear is whether other genetic variants associated with individual diseases also form common pathways/mechanisms for autoimmunity. Recent results from GWA studies suggest that common genetic mechanisms may underlie the observed clustering of multiple autoimmune diseases within a person or family. In this work we have tested the hypothesis that immunologically relevant genetic variation will either (1) underlie risk to all immune-mediated diseases, implicating a global immunological process; (2) influence risk to a discrete subset of diseases, implicating molecular entities underlying that co-morbidity; or (3) modulate risk for only one disorder thereby implying a disease-specific process.

A central goal of complex disease genetics is to uncover the pathways perturbed in disease and shed light on the underlying cellular processes. Despite a wealth of molecular insight into immune function few key pathways underlying genetic susceptibility to immune-mediated diseases have been elucidated. To identify these processes in immune-mediated inflammatory and autoimmune disease, we tested genetic variation contributing to seven diseases. We observed an overwhelming abundance of commonality across these phenotypes, assorting into cohesive phenotype-genotype groups that appear to underlie co-morbidities. By analyzing loci known to associate to at least one disease, we are able to identify groups of diseases that should be considered as a unified phenotype and analyzed together. We further demonstrate that this approach generates novel biological insights into pathogenesis, often difficult to obtain from genomic studies of single traits [20].

We have described a novel statistic, CPMA, which assesses evidence for multiple associations to a marker. Rather than perform a meta-analysis, which would only detect association to *all* phenotypes (or suffer from heterogeneity) or test all combinations of phenotypes which would increase the multiple testing burden, we look for deviation in the distribution of association *p* values. Our statistic thus detects markers associated to at least some, but not necessarily all, phenotypes; we note that this is a single degree of freedom test, providing high power to reject the null hypothesis. This power comes at the price of not knowing to which phenotypes the marker is associate; we overcome this with our clustering analysis, which resolves groups of markers associating to the same diseases. Thus our analytic strategy is able to both detect shared associations and identify the relevant phenotypes.

Our approach appears capable of distinguishing distinct genetic effects in the same locus in addition to validated shared associations. For example, it is now clear that the two signals in the *IL2/IL21* locus on chromosome 4q27 are distinct, with T1D mapping to *IL2* and other diseases to *IL21* [21]. Our analysis detects this difference, clustering the two SNPs representing these associations separately (Figure 1, labeled “IL2” and “IL2/IL21”, respectively). Conversely, previous reports of an overlap in association between T1D and celiac disease [15] were in regions encoding genes highly expressed in T lymphocytes (*RGS1*, *PTPN2* and *CTLA4* in celiac; *PTPN2* and *CTLA4* in T1D). Our analysis identifies all these regions as CPMA-positive and highlights the second associations in T1D and celiac shown by Smyth *et al.* [15], indicating that our approach could be used to prioritize marginal associations for replication. We also observe other potential associations. For example, rs2816316 on near *RGS1* exhibits evidence of association to MS; rs2542151 and rs1893217 on near *PTPN2* has modest association to psoriasis. These last observations, whilst suggestive, require further investigation given the known effects of these regions on other diseases.

In summary, our multi-disease approach is applicable beyond the immune-mediated inflammatory and autoimmune diseases, to current studies of related traits in pharmacology, metabolic and psychiatric disease and in genetic studies of cellular phenotypes such as gene expression. For most studies of the genetic basis of complex human phenotypes, the pathogenic processes are still far from understood and biological pathways may be identified using these methods. Ultimately, these results will contribute to an improved molecular nosology of mechanistic definitions and, ultimately, towards improving clinical care and human health.

## Materials and Methods

### Ethics statement

All data were drawn from previously published genome-wide association studies from consortia with appropriate ethics oversight from their respective institutional review boards. As only summary data from a small number of markers across the genome were used here no further ethical issues arise.

### Patient cohorts

Data were obtained from previously described case/control GWA studies of celiac disease [22], Crohn's disease [2], multiple sclerosis [5], psoriasis [6], rheumatoid arthritis [7], systemic lupus erythematosus [23] and type I diabetes [24] as shown in Table 1. We note that, with the exception of psoriasis, in these cohorts diagnosis of a second immune-mediated disease is a criterion for exclusion, thereby minimizing co-morbidity as a source of bias in our study.

### Locus selection

For our analysis we selected 140 independent SNPs (*r^2^*<0.2) with reported associations to an immune-mediated disease in a genome-wide association scan and replicated in independent samples in that disease to combined genome-wide significance [25]. We then chose proxies for those SNPs present on the major versions of Affymetrix and Illumina genome-wide genotyping platforms [26]; 107 SNPs had sufficient data coverage to be included. Where possible we used the SNP originally reported; if data were unavailable for that marker, we chose a high LD proxy (HapMap/CEU *r^2^*>0.9) to represent the region.

### Cross-phenotype meta-analysis

Our CPMA analysis relies on the expected distribution of p-values for each SNP across diseases. Under the null hypothesis of no *additional* associations beyond those already known, we expect association values to be uniformly distributed and hence *-ln(p)* to be exponentially decaying with a decay rate λ = 1. We calculate the likelihood of the observed and expected values of λ and express these as a likelihood ratio test:
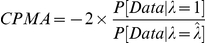



This statistic therefore measures the likelihood of the null hypothesis given the data; we can reject the null hypothesis if sufficient evidence to the contrary is present. We note that, because we only estimate a single parameter, our test is asymptotically distributed as 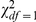. This gives us more statistical power than relying on strategies combining association statistics, which would consume multiple degrees of freedom.

### SNP–SNP distance calculation and clustering

To compare the patterns of association for multi-phenotype SNPs we first calculate SNP-SNP distances and then use hierarchical clustering on that distance matrix to assess relative relationships between SNP association patterns.

Calculating distances based directly on *p* values or the underlying association statistics is problematic, as each contributing study has slightly different sample sizes and therefore different statistical power to detect associations. Thus, distance functions based on numeric data – which incorporate magnitude differences between observations – would be biased if studies have systematically different data. Normalization procedures can account for such systematic differences but may fail to remove all bias. To reduce the impact such systematic irregularities might have on our comparison, we bin associations into informal “levels of evidence” categories. We define four classes (1<*p*<0.05, 0.05<*p*<0.001, 0.001<*p*<1×10^-6^, 1×10^−6^<*p*) and thus reduce our data to a SNP x disease matrix where entries are categorical variables describing these classes. We then calculate the Euclidean distances between pairs of SNPs using Gower's method for categorical data [27], which accounts for the discrete nature of the data.

To compare the distance relationships between SNPs we use hierarchical agglomerative clustering. This process joins single entities (in this case, SNPs) or groups of entities together if certain criteria are met. Successive rounds of clustering are preformed in an iterative way until all groups are joined, resulting in a tree of relationships where similar entities cluster on the same branches. In this analysis we cluster SNPs based on the Gower distance matrix using Ward's method for joining entities [28]. In contrast to linkage clustering methods, Ward's method seeks to minimize the information lost during the clustering process, calculated as the error sum of squares (ESS). The higher the ESS the more information is being lost due to inaccuracy of grouping entities together. This method thus seeks compact, spherical clusters of data which are maximally similar.

All distance and clustering analysis was done using the *StatMatch* and *stats* packages in the **R** programming language [29].

### Cumulative association statistics

We compute per-cluster, per-disease cumulative association statistics by combining *p* values using Fisher's omnibus test, where the cumulative statistic *S_cum_* on *N p*-values is defined as:
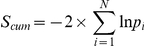



and *S_cum_* follows the 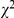 distribution with *2N* degrees of freedom.

### Protein–protein interaction analysis

We use previously described methodology [19] to assess whether proteins encoded around SNPs in each cluster interact. Briefly, we first compile lists of all proteins that an association may affect by defining locus boundaries around each SNP in terms of linkage disequilibrium and including all proteins overlapping this region. We then use a high-confidence protein-protein interaction map ([30] as modified in [19]) to ask whether proteins encoded around SNPs in each cluster interact either directly or via a common intermediary and assess the significance of such observations relative to the local structure of the protein-protein network as described elsewhere [19], using 4000 permutations. These data and methodology are publicly available for download and via a webserver (http://www.broadinstitute.org/mpg/dapple).

## Supporting Information

Dataset S1 Complete SNP-wise association data. Here we present the complete dataset on which we base our analysis. All data have been previously published as detailed in the main manuscript and in the key below, and are based on chi-square (1 df) or Z association statistics. Where not provided, we computed Z scores as the square root of the cognate chi-squared statistic. Sign was assigned with reference to the minor allele declared in the psoriasis GWAS (chosen arbitrarily). SNP - marker name. CHR – chromosome. POS - physical position (hg18). major_al - major SNP allele. minor_al - minor SNP allele. RA.Z - association Z score for rheumatoid arthritis (Stahl *et al*.Nat Genet 2010) [7]. RA.P - association p value for rheumatoid arthritis (Stahl *et al*.Nat Genet 2010) [7]. PS.Z - ditto for psoriasis (Nair *et al*. Nat Genet 2009) [6]. PS.P - ditto for psoriasis (Nair *et al*. Nat Genet 2009) [6]. MS.Z - ditto for multiple sclerosis (De Jager *et al*. Nat Genet 2009) [5]. MS.P - ditto for multiple sclerosis (De Jager *et al*. Nat Genet 2009) [5]. SLE.Z - ditto for systemic lupus erythematosus (Gateva *et al*. Nat Genet 2009) [23]. SLE.P - ditto for systemic lupus erythematosus (Gateva *et al*. Nat Genet 2009) [23]. CD.Z - ditto for Crohn's disease (Barrett *et al*. Nat Genet 2008) [2]. CD.P - ditto for Crohn's disease (Barrett *et al*. Nat Genet 2008) [2]. CeD.Z - ditto for celiac disease (Hunt *et al*. Nat Genet 2008) [4]. CeD.P - ditto for celiac disease (Hunt *et al*. Nat Genet 2008) [4]. T1D.Z - ditto for type I diabetes (Barrett *et al*. Nat Genet 2009) [24]. T1D.P - ditto for type I diabetes (Barrett *et al*. Nat Genet 2009) [24]. Disease - disease in which the SNP was originally reported: AITD autoimmune thyroid disease; AS ankylosing spondylitis; BD CD Crohn's disease; MS multiple sclerosis; PS psoriasis; SLE systemic lupus erythematosus; T1D type 1 diabetes; UC ulcerative colitis. cpma.p - p value for CPMA statistic (chi-squared, 1 df). Genes - nearby notable genes.
(TAB)

Figure S1 Enrichment in immune tissue expression for interacting genes encoded close to SNPs in (A) cluster 1 and (B) cluster 4. Following Rossin *et al*.[19] we looked for preferential expression of significant network genes in tissue subsets. Of the genes encoded around SNPs in clusters 1 and 4 (as defined in Figure 1), we found that those participating in significant networks are enriched in expression (purple circles) in immune tissues (red bars). Other genes encoded around those SNPs are not enriched in the same tissues (black circles). Thus interacting genes encoded around SNPs associated to the same immune diseases are preferentially expressed in immune tissues. Interacting genes for the remaining significant group, cluster 2, were not enriched.
(PDF)
