# Supplementary figures and images for: Pervasive Sharing of Genetic Effects in Autoimmune Disease
Source: PLoS Genet. 2011 Aug 10;7(8):e1002254. doi: 10.1371/journal.pgen.1002254 (PMC3154137; doi:10.1371/journal.pgen.1002254)

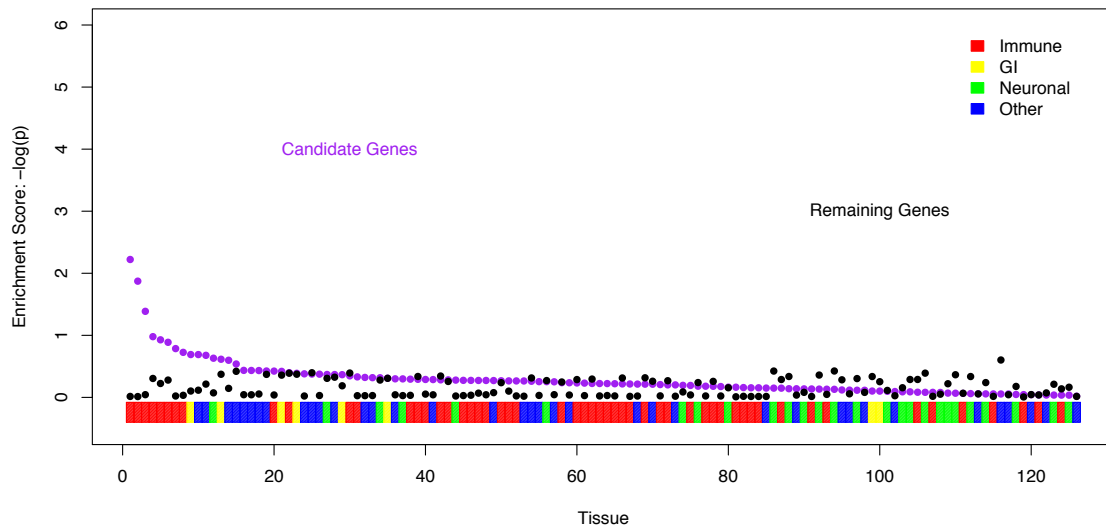

A

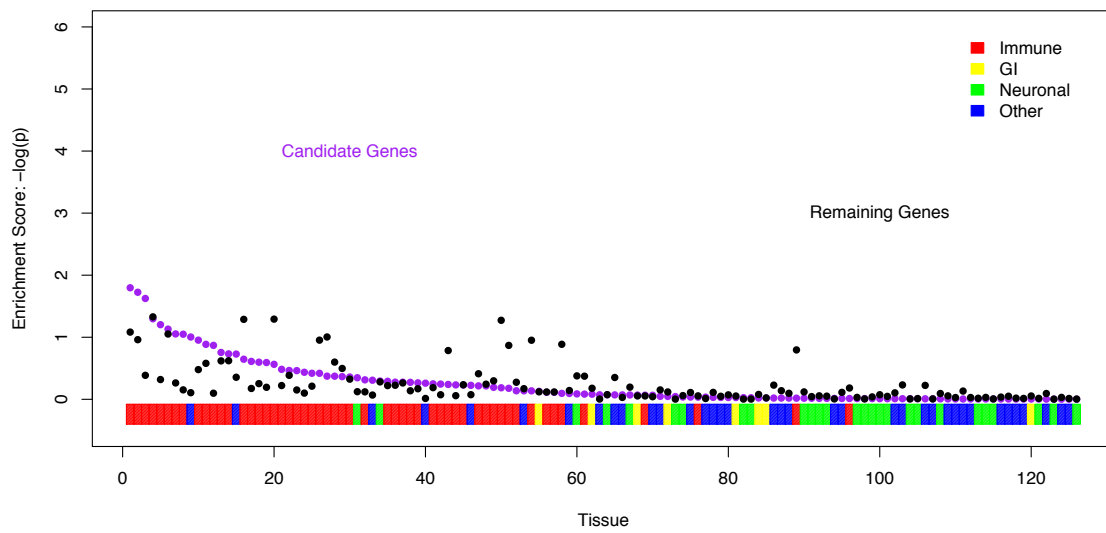

B

Supplement: Figure S1 — Enrichment in immune tissue expression for interacting genes encoded close to SNPs in (A) cluster 1 and (B) cluster 4. Following Rossin et al.[19] we looked for preferential expression of significant network genes in tissue subsets. Of the genes encoded around SNPs in clusters 1 and 4 (as defined in Figure 1), we found that those participating in significant networks are enriched in expression (purple circles) in immune tissues (red bars). Other genes encoded around those SNPs are not enriched in the same tissues (black circles). Thus interacting genes encoded around SNPs associated to the same immune diseases are preferentially expressed in immune tissues. Interacting genes for the remaining significant group, cluster 2, were not enriched. (PDF) [file pgen.1002254.s002.pdf]
